# Impact of the shedding level on transmission of persistent infections in *Mycobacteriumavium* subspecies *paratuberculosis* (MAP)

**DOI:** 10.1186/s13567-016-0323-3

**Published:** 2016-02-29

**Authors:** Noa Slater, Rebecca Mans Mitchell, Robert H. Whitlock, Terry Fyock, Abani Kumar Pradhan, Elena Knupfer, Ynte Hein Schukken, Yoram Louzoun

**Affiliations:** Gonda Brain Research Center, Bar-Ilan University, Ramat Gan, Israel; Department of Population Medicine and Diagnostic Sciences, Cornell University, Ithaca, NY USA; Department of Mathematics and Computer Science, Emory University, Atlanta, GA USA; New Bolton Center, University of Pennsylvania, Kennett Square, Philadelphia, PA USA; Department of Nutrition and Food Science, Center for Food Safety and Security Systems, University of Maryland, College Park, College Park, MD USA; Utrecht University, Utrecht, The Netherlands; GD Animal Health, Deventer, The Netherlands; Department of Animal Sciences, Wageningen University, Wageningen, The Netherlands; Department of Mathematics, Bar-Ilan University, Ramat Gan, Israel

## Abstract

Super-shedders are infectious individuals that contribute a disproportionate amount of infectious pathogen load to the environment. A super-shedder host may produce up to 10 000 times more pathogens than other infectious hosts. Super-shedders have been reported for multiple human and animal diseases. If their contribution to infection dynamics was linear to the pathogen load, they would dominate infection dynamics. We here focus on quantifying the effect of super-shedders on the spread of infection in natural environments to test if such an effect actually occurs in *Mycobacteriumavium* subspecies *paratuberculosis* (MAP). We study a case where the infection dynamics and the bacterial load shed by each host at every point in time are known. Using a maximum likelihood approach, we estimate the parameters of a model with multiple transmission routes, including direct contact, indirect contact and a background infection risk. We use longitudinal data from persistent infections (MAP), where infectious individuals have a wide distribution of infectious loads, ranging upward of three orders of magnitude. We show based on these parameters that the effect of super-shedders for MAP is limited and that the effect of the individual bacterial load is limited and the relationship between bacterial load and the infectiousness is highly concave. A 1000-fold increase in the bacterial contribution is equivalent to up to a 2–3 fold increase in infectiousness.

**Electronic supplementary material:**

The online version of this article (doi:10.1186/s13567-016-0323-3) contains supplementary material, which is available to authorized users.

## Introduction

The phenomenon of super-shedding, where a small fraction of individuals contributes a disproportionate load of infectious pathogens to the exposure experience of susceptible individuals, has received much attention in the past several years with respect to human disease [1] and selected livestock pathogens, as well as the virtual spread of viruses [2–5]. Super-shedding individuals have been reported with infections, such as *Escherichia coli* O157: H7 [6], paratuberculosis [7, 8], HIV [9], influenza [10], and *Salmonella* [11]. However, the precise contribution of individuals producing a high concentration of pathogens to infection dynamics in the population remains poorly understood.

If infection is a direct host-to-host contact process, even with all contacts being effective (i.e. every contact with a susceptible results in transmission of infection), the maximum contribution of each infectious individual is capped by contact rates. However, if infection is driven by indirect contacts where susceptible individuals are exposed to infectious organisms present in a well-mixed environment, and the contribution of an individual scales linearly with the amount of pathogen shed, individuals shedding pathogens several orders of magnitude higher than the average infectious individual would drive infection dynamics by increasing the total force of infection by a similar order. Conversely, removing these highly infectious individuals would cause a dramatic reduction of pathogen concentrations and a virtual elimination of new infections. Indeed, strategies to eliminate high shedders have been proposed as a method of preventing infections [12]. However, endemic infections persist in the presence as well as in the absence of super-shedders [13, 14]. Assume for example that a super-shedder produces 1000 times more infective doses than a regular infected host, and that one in 100 infected hosts is a super-shedder. If the force of infection would have been linear in the pathogen load, one would obtain an approximate 10-fold increase in the force of infection in the presence of super-shedders in comparison with the same infection in the absence of super-shedders. This 10-fold larger force of infection would result in very large outbreaks. However, such an increase in infection prevalence is generally not observed or expected [13, 14], leading to the so-called super-shedder paradox: an observed high amount of shedding that results in relatively little impact on infection dynamics. This unexpected limited correlation between infectious burden and force of infection may be hypothesized to be due to transmission models where the force of infection is a concave function of the pathogen load rather than a linear function. Indeed, previous works have proposed multiple types of non-linear relationships between the force of infection and the pathogen load [15, 16]. Among those, the simplest is probably a power relationship [17], as is also used here.

We here study a detailed data set of an endemic infectious disease to test our hypothesis that transmission models have a significantly sub-linear (concave) relationship between the pathogen load and the force of infection.

**Figure 1:**
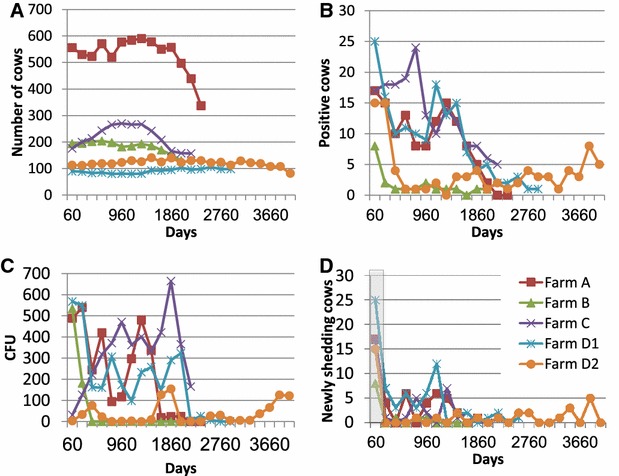
**Description of data on the five farms.** **A**. Number of cows with at least one adult sampling time point in each farm per day. (Because heifers are only eligible for testing at first calving, the number of cows appears lower for the last two sampling time points). **B**. The number of cows with positive samples per day. **C**. Total CFU for all cows per day. **D**. Number of cows which started shedding per day. The first sample day was not taken into account, since we had no earlier information and could not determine when the first appearance of clinical signs (high shedding) occurred. While some farms vary over time and contain information on the dynamics of the epidemics, others, such as Farm D2, have a very flat fraction of infectious cows and are thus not informative.

We use longitudinal data from populations of dairy cattle endemically infected with a single persistent infection with the organism *Mycobacteriumavium* subspecies *paratuberculosis*, or MAP. Infection with MAP occurs usually in early life through oral intake of pathogens. The infection establishes itself in the intestinal tract and eventually adult animals shed the organism in fecal material [18]. Individual infected animals have a wide distribution of infectious shedding loads, ranging upward of five orders of magnitude ([8] and Figure 1). In the context of environmentally transmitted diseases, the term super-shedder has been loosely defined and is proposed for animals shedding at the high end of the distribution of shedding loads [8].

A parallel term for super-spreader has been proposed for individuals that contribute to a larger number of new infections in biological diseases, as well as for virus spreading in networks [19–25]. The super-spreaders are also treated as hubs, which drastically increase the spread rate in contact networks. Such super-spreaders could be the result of a high number of contacts (e.g. in sexually transmitted diseases), the result of a high pathogen dose in the super-spreader, or the result of a highly virulent pathogen. We here argue that in the studied case, super-shedding does not induce super-spreading, and use a maximum likelihood framework to demonstrate this argument.

Information on MAP shedding progression is available for experimentally and naturally infected animals [26–28]. Mathematical models that capture transmission dynamics of MAP have been developed for dairy animals in the US and EU [13, 29–32] using deterministic and stochastic frameworks. The majority of such models rely on an assumption of direct transmission via the fecal-oral route; however, in some models of paratuberculosis transmission, animals contribute infectious material to a common environment and this environment serves as an indirect source of transmission [31, 33, 34]. The dominant transmission strategy for MAP is not well known, and the distinction between the two transmission pathways, direct or indirect, remains poorly elucidated.

The rate of being born into the MAP infected state has been estimated to be as high as 15% of all newborn calves; a more precise estimate from the farms in our data was obtained from detailed molecular typing of isolates known to have infected both the dams and the daughters (see [12] for detailed information). Transmission based on confirmation with molecular typing of the isolated MAP organisms was estimated as approximately 1% incidence of vertical transmission among all pregnant dams, and in their farms with an approximate 25% prevalence of infection in adult animals, a 4% incidence of vertical transmission among known MAP infected dams. Hence, in the data used in our models, vertical transmission was not the dominant route of transmission and was ignored here.

We here develop a parameter estimation method to partition infection transmission into contributions from direct contact, indirect contact and an unmeasured background transmission. The estimate allows us to capture the influence of individual shedding patterns on overall transmission dynamics. We then show using this model that at least in the case studied here, super-shedding does not induce super-spreading.

## Materials and methods

### Experimental observations

We analyze MAP shedding in adult animals that calved at least once on four dairy farms (A, B, C, D). Two of these farms were located in Pennsylvania, one in Vermont and one in New York. The animals are outdoors. From all cows older than 2 years of age, fecal samples were collected at least twice a year. Fecal samples were processed using the double incubation method, 2 gms were placed into a 50 mL conical tube containing 35 mL of water. The sample was rocked for 30 min and then allowed to stand at room temperature for 30 min. Then 5 mL was transferred from the top of the tube and placed into a 50 mL conical tube containing 25 mL ½ strength BHI/0.9% HPC, these were incubated overnight at 37 °C. The samples were centrifuged for 30 min at 900 *g*, decanted and then the pellet was resuspended with 1 mL of antibiotic brew (Amphotericin B, Naladicix Acid, Vancoymcinin ½ strength BHI). Samples were then incubated overnight at 37 °C. Following incubation, four tubes of Herrold’s egg yolk media (BD Diagnostics) were inoculated with 0.2 mL per tube and then incubated with loosened caps in a slanted position at 37 °C. At 2 weeks the lids were tightened and the tubes placed into an upright position. The tubes were read every 2 weeks with the final reading and colony counting at 16 weeks. Total sum of colony forming units (CFU) across four tubes was multiplied by 5.3 to determine CFU of MAP/g of feces. Classically a bacteria count of more than 10^4^ CFU/g has been used as the definition of super-shedding [35]. However, since the current analysis is based on explicit quantitative measurements of the shedding level, such a definition is not required for the current analysis. All fecal isolates collected from the farms were stored for future analyses. In one of these farms (A), additional information on available bacterial strain types was used (14 types). The typing method applied here is a restricted subset of a multi-locus short-sequence-repeat method as previously published (Loci 1, 2, 8, 9, 10) [36]. Here, we used the two most frequent strains for an individual strain analysis, as will be further discussed. Three farms (A, B, C) have also detailed MAP ELISA data and tissue culture information from a subset of slaughtered animals [37]. One farm (D) has long term recordings of bacterial load (over 20 years) from all adult cows as previously described [14]. In farm D, the sensitivity of detection increased once in the 20-year period due to the introduction of an improved diagnostic test. We, thus, separated the samples for this farm into two datasets (D1, D2), with D1 using the initial diagnostic test and D2 following application of the new testing method. All farms received all MAP culture and ELISA data and were able to use this information to improve their MAP control plans. Further detailed descriptive information on these farms appears in Additional file 1 and in previous publications [14, 38]. Obviously in some experiments, we may miss a shedding cow. However, we assume that with repeated testing over time, especially on animals with post-mortem testing, we accurately identify infected cows. We also assume that the actually observed CFUs of MAP are a reflection of the true infectiousness of an infected animal. This may not be the true level of CFU of MAP, but it would represent the relative infectiousness of the infected host. Note that if there is a sub-estimate of the CFU level, this would mainly affect high CFU levels due to right censoring in the ability to quantify bacterial load on a culture slant. Thus our estimate of gamma is an upper bound, and the effect of super-shedders may be even more limited than proposed by the current analysis.

### Farm descriptions

Over the course of the study, Farm A had 1044 cows available for analysis, whereas the other populations (Farms B and C, D1 and D2) had between 242 and 385 cows (Additional file 1, about 98% of the cows were continuously sampled for shedding). The duration of the period examined in each population was between 6–11 years. For each farm, there were between 1222 and 6404 fecal samples and between 12 and 70 initial shedding events. The numbers of positive ELISA tests were: 43 in farm A, 5 in farm B, and 20 in farm C. The numbers of animals with positive post-mortem samples were: 66 in farm A, 13 in farm B, and 30 in farm C. For farm D, no ELISA or post-mortem samples were collected, and in this farm all analyses were based on fecal sample results. The number of newly infected cows and the pathogen load varied over time in the different farms (Figure 1). This variation may be used to estimate the parameters of the infection. More information on the farms can be found in [12, 39].

### Extrapolation

**Figure 2:**
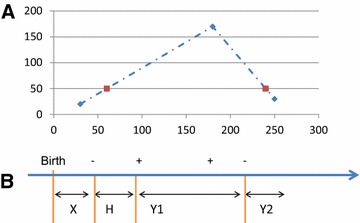
**Schematic figure.** **A**. Example of interpolation. Blue squares represent the original data (whenever it is available). Dashed blue line is the interpolation and red squares represent the new “samples” in the new time points (every 180 days). **B**. Schematic timeframe of positive and negative fecal data for one cow. “−” and “+” indicate negative and positive samples, respectively. In this example, there are two negative and two positive samples. The cow is susceptible (X) until the first negative sampling point, latent (H) between the first negative sampling and the first positive sampling, late shedding (Y1) between the first positive sampling and the second negative sampling and non-shedding infected (Y2) between the last negative sampling and its death.

The samples provided were taken in unequal intervals of approximately half a year apart. In order to simplify the formalism, we aligned the time schedule such that day 60 in the original data is the first time step, and the following time steps occur at constant intervals of 180 days (statistics for the original data and the “interpolated” data can be found in Additional file 2). The total number of observations in the original and aligned datasets are similar; and for the vast majority of 180 days intervals, at least one observed data point was available. We interpolated the fecal amounts of the original data to the aligned time points for all the cows. For example, assume a sample of 20 CFU at day 30 and a following sample of 170 CFU at day 180, and then a sample of 30 CFU at day 250. We used linear interpolation to calculate the amount of CFU for all the days in between. We then estimated the parameters explaining the dynamics using the CFU values at days 60, 240 etc. In the above case, the “sampled” value on day 60 will be 50 CFU and on day 240 it will be also 50 CFU. A graphical representation of the observed infection patterns is given in Figure 2A.

### Infection progression description

In order to estimate the parameters of the infection, we simplified previous state-transition models [13] to three states (Figure 2B; see Additional file 3 for all symbols used): susceptible (X), latent (H), shedding (Y1), and allow a fourth non-shedding infected state (Y2). We combined initial early shedding and latent periods into one compartment (H). This H compartment was treated as uniformly non-infectious for each optimization. Since published experimental data [40–42] and field data [37] indicate that animals are not consistently detectable by fecal culture following initial shedding (Y1), we allowed animals to exit this shedding phase into a non-shedding (Y2) period. Other models were tested with sequentially broader assumptions of the size of the infectious population (see Additional files 4 and 5 for more details) but no real differences were seen. Definition of “infected” cows is the same.

We assumed that the latent period H has a length distribution *f*(Δ*t*), where Δ*t* is the time interval from infection at time *i* to the appearance of detectable shedding in individual animals (Y1) at time *l*. We used a Gaussian model (Additional files 4 and 6) for estimating the duration of the latent period. We also tested an exponential model, representing a constant probability of moving from H to Y (Additional file 4), but this alternative model did not perform better than the Gaussian model (Additional file 7). Note that these models are formulated here only for the parameter estimation using the maximum likelihood method, and are not simulated nor solved by ODEs. The model is used to define the likelihood function of an observed farm shedding pattern.

Due to testing intervals of approximately 6 months and left censoring resulting from the initiation of sampling at the start of lactation, usually at the age of 2 years, observed shedders can begin shedding before the time of detection and may keep shedding for an unknown period until the next sampling time point. We assume that animals that start shedding are both infectious (according to the appropriate model) and infected. We further assume that animals shedding MAP in consecutive sampling time points are shedding in the interim period. Animals that switch shedding states from one sampling time point to the next are assumed to have initiated or ceased shedding at the midpoint between those two measurements. We assign the population exit date at 90 days (half the sampling interval) past the last sampling time point for all individuals.

### Sources of infection

We take into account three potential sources of infection: a constant background infection pressure unrelated to infectious animals or bacterial shedding (*δ*), direct transmission through infected individuals (*β*), and indirect transmission via shed bacteria (*α*). These three sources have the following contributions to infectivity:

- A probability for a susceptible cow to get infected from the constant background infectivity term (*δ*), which can represent unmeasured sources like inflow of bacteria from other farms, does not depend on the amount of MAP bacteria being shed or on the number of MAP-infected cows in the herd.
- The cow-to-cow direct infection term (*β*) is based on the assumption that each herd is well mixed and that transmission is direct, and that the contribution of each infected cow is not affected by the total number of cows. This term is proportional to the number of infected cows (a density dependent transmission) which is denoted by *y*. This value is obviously time dependent.
- The indirect infection term (*α*) is based on the assumption that transmission is proportional to the available MAP bacterial burden (*w*). The force of infection is then proportional to the amount of bacteria raised to the power *γ* [denoted by *αw*(*γ*)]. This term is also time dependent. Specifically, the probability of being infected is proportional to a power of the single cow’s total MAP amount $w\left( \gamma \right) = \sum\nolimits_{i \in cows} {w_{i}^{\gamma } }$. We have also tested an alternative model where the infection is proportional to the total amount of free bacteria to the power of *γ*: $w\left( \gamma \right) = (\sum\nolimits_{i \in cows} {w_{i} )^{\gamma } }$, as discussed in the Additional file 4. This alternative model did not produce better results. The persistence/survival of the bacteria in the environment is of limited practical importance, since we use intervals of 180 days, and the bacteria survives typically less than 6 months in the environment.

The total force of infection is given by Equation (1):

1 $$f_{t} = \delta + \beta y(t) + \alpha w\left( {\gamma ,t} \right) .$$

In all following equations, we will assume a dependence of the number of infected cows, infection probability and amount of shed bacteria, also when not explicitly stating the time dependence.

### Maximum likelihood fit

We tested the fit of the observed shedding patterns to the models above, using a maximum likelihood (ML) binomial model. The probability of the observed infection pattern is defined as its likelihood (Equation 2), where for each day *y*_*l*_ is the number of cows that can potentially begin shedding on timestep *l*; *S*_*l*_ is the number of susceptible cows on timestep *l*. If there are *S*_*l*_ susceptible cows and a probability *p*_*l*_ of starting to shed on timestep *l*, then there is a probability of *P*(*y*_*l*_) to observe *y*_*l*_ cows which started shedding on timestep *l*. This probability is multiplied across all timesteps of the analysis in order to get the total probability of observing the observed infection pattern *P*(*Y*) (Equation 2):

2 $$P(Y){ = }\prod\limits_{1 < l} {P(y_{l} )} = \, \prod\limits_{1 < l} {\left( {\begin{array}{*{20}c} {S_{l} } \\ {y_{l} } \\ \end{array} } \right)} p_{l}^{{y_{l} }} \left( {1 - p_{l} } \right)^{{(S_{l} - y_{l} )}}$$

The fit of a model to the observations was computed using the log likelihood of the observed shedding patterns, using the infection pressure from the observed infected or shedding animals. The best fit can be computed by maximizing the log-likelihood (Equation 3):

3 $$\begin{aligned} {\text{LL}} =& \sum\limits_{1 < l} {y_{l} } \log (p_{l}^{{}} ) + (S_{l} - y_{l} )\log \left( {1 - p_{l}^{{}} } \right) + \sum\limits_{1 < l} {\log \left( {\begin{array}{*{20}c} {S_{l} } \\ {y_{l} } \\ \end{array} } \right)} \hfill \\ =& \sum\limits_{1 < l} {y_{l} } \log (p_{l}^{{}} ) + (S_{l} - y_{l} )\log \left( {1 - p_{l}^{{}} } \right) + C \hfill \\ \end{aligned}$$

Note that the term $$\left( {\begin{array}{*{20}c} {S_{l} } \\ {y_{l} } \\ \end{array} } \right)$$ is not affected by the parameters of the model, and is ignored in all following computations.

Infected cows are only detected when they start shedding. These shedding patterns can occur years after the infection. In order to take into account the delay between the time of infection and the observation of shedding patterns, we convolute the force of infection *f*_*t*_ (Equation 1) with a forward Gaussian with an average of *µ* and a standard deviation of *σ* (Equation 4), and calculate the probability (*p*_*l*_) for a cow to start shedding on timestep *l* as 1 minus the probability that it did not shed (Equation 5).

4 $$Z_{l} = \sum\limits_{t} f_{t} \frac{1}{\sqrt{2\pi}\sigma}e^{-\frac{\left( {l - t - \mu } \right)^{2}}{2\sigma ^{2}}}$$

5 $$p_{l} \, = \, 1 - e^{{ - Z_{l} }}$$

The first sampling time point for each farm was not considered, since there was no reliable information on the time when the cows that started shedding in this first sample were first infected (Figure 1D).

When analyzing a single farm, the score obtained from the optimization is simply the log likelihood as in Equation 3. When analyzing all the farms together, the total score is the sum of the costs for each individual farm. The cost is minus the maximum likelihood. Thus, a lower cost is a better likelihood. A better fit for a model with a set of parameters (higher likelihood) represents a better solution. A numerical optimization (Nelder-Mead [43] with 1000 random initial conditions) is performed in Matlab to find the parameter set producing the highest log likelihood. We first fit the data of each farm separately with its own set of parameters *α*, *β*, *γ*, *δ*, *μ*, *σ*. For each farm we obtained a different cost.

We then tested for the optimal score, when we set all parameters (*α*, *β*, *γ*, *δ*, *μ*, *σ*) to be equal among all farms. Such an assumption provides an average contribution of each infection term in each farm: the cow-to-cow direct term, the indirect term via bacteria shed and the constant term.

In order to compare between the different models, we used the likelihood ratio test [44]. We calculated the test statistic which is twice the log of the likelihoods ratio (or twice the difference in log-likelihoods). We then calculated the Chi squared value of that difference with the number of degrees of freedom between the two models, and checked significance.

### Contribution of each transmission route to the total infectivity

Once the best parameters were obtained, we analyzed the contribution of each term of transmission to the total force of infection for each farm. This was done by calculating the average value per day of the indirect transmission term [*αw*(*γ*)] and of the direct cow-to-cow transmission term (*β*). The constant infection pressure contribution is given simply by the value of *δ*.

### Strain-level analysis

**Figure 3:**
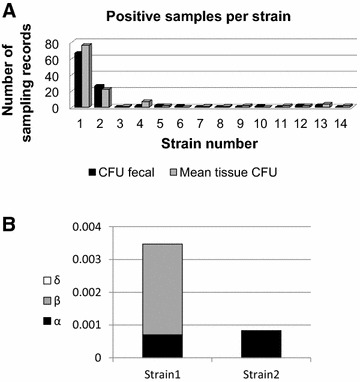
**Strains information and results. A**. Total number of observations per strain in Farm A. Only strains 1 and 2 have more than 10 positive samples. **B**. Contribution results for individual-strain analysis. Contribution of each term in the model (single cow non-linear) to the average infectivity for each strain for farm A.

Beyond the difference in shedding patterns, pathogens have a genetic variability. In order to test the effect of this variability on the model, we analyzed the disease dynamics using strain specific information available for farm A. In this farm, the MAP bacteria were typed to identify individual MAP strains as defined above. Strain typing was done using multi-locus short sequence repeat sequencing [36]. Due to paucity of individual data points for less common strains, we separately evaluated infection dynamics of the two dominant strains, which represent 91% of all cow-strain information recovered (Figure 3A).

### Simulation

In order to correlate the parameters above with optimal culling, we simulated the farms dynamics and extracted the number of MAP infected animals in steady state, assuming different culling strategies. We simulated a farm with N cows (N being the average number of alive cows in all five farms together). For each interval, we calculated the probability for a cow to get infected using the equation:

6 $$P({\text{cow gets infected)}} = \alpha *TotalBacteriaShed^{\gamma } + \beta *NumberOfSheddingCows + \delta$$

On the first day, we chose 20 cows to start shedding. The initial number of shedding cows had no effect on the steady state results. Every infected cow starts shedding *µ* intervals after the initial “infection”. It continues shedding until it reaches the cutoff value and then dies. In addition, every cow dies at the “age” of 7 intervals (3 ½ years), which is the mean lifetime of cows in the real farms. The shedding level distribution is taken from the observed shedding level distribution (Additional file 2). When a cow dies, another cow is born to maintain a constant herd size. The simulation was performed for 350 intervals (each representing a period of 180 days). We calculated the average number of MAP infected cows and the average number of culled cows in equilibrium (after the first 50 intervals). The number of infected cows was averaged among 200 realizations of the simulation. We computed the number of infected and culled cows as a function of the culling level (the shedding level above which cows are culled).

## Results

### Comparison between sources of infection in each farm separately

**Figure 4:**
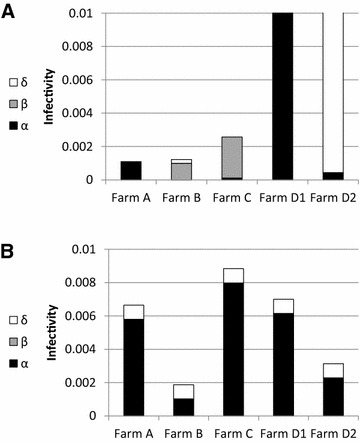
**Contribution of each term in the models to the average infectivity.** **A**. Contribution of each term in the models to the average infectivity in each farm, when optimization was done separately on each farm. The first term (*α*) is infection by free (externally sourced) bacteria. The second term (*β*) is cow-to-cow infection and the last term (*δ*) is a constant source. **B**. Contribution of each term in the model to the average infectivity for each farm when using one set of parameters for all the farms. The main contribution was from the indirect transmission term, and the secondary infectivity is the constant term. There was no contribution from the direct transmission term. The contributions of each term differ among farms, since each farm has a different fraction of infected cows. However, most models yield similar results in the same farm.

In order to estimate the model that best explains the observed shedding patterns, we compared models with constant infection pressure to models with additional direct transmission and indirect transmission terms. In each farm, we evaluated the parameters that best explain the epidemic spread, using multiple possible infection models and different definitions of infectious cows. Alternative models to the one presented in the main text are detailed in Additional files 4, 8 and 9. None of the alternative models provided a higher likelihood than the model presented here. Multiple models had a likelihood that was not significantly different from the null model. However, all the optimal (maximal) likelihood solutions belonged to one of the following two possible solution types: (A) An infection model where direct infection is the main source of infection. In such a model the amount of bacteria shed by each cow does not influence transmission from that animal; (B) A model where indirect infection is the main transmission route, but the power relating shedding levels and infectiousness *γ* is between 0 and 0.69 (Table 1). In other words, the amount of bacteria shed increases infectiousness, but the effect was sub-linear (i.e. doubling the shed bacteria leads to much less than a doubling in the force of infection). See Table 1 for the optimal values in each farm.) Farm D2 had a constant number of new infections and flat pathogen loads and was thus not informative and could not be differentiated from a constant infectivity even if direct or indirect transmission were potentially driving infection (Figures 1 and 4A). One can thus summarize that in all plausible models, the effect of the shedding level of each cow is limited. However, from the single farm analysis, one cannot conclude whether this is the result of direct transmission, or indirect transmission with a highly concave transmission probability.

**Table 1:** **Parameters obtained for the best fit.**

	Cost	*α*	*µ*	σ	δ	γ	β
Farm A	170.1717	3.49E−05	1	0.100166	0	0.551659	0
Farm B	28.042	0	2.8563	0.16409	0.000227	0.19292	0.00029
Farm C	113.82	6.59E−06	1.0064	0.13209	0	0	0.000138
Farm D1	178.2996	0.002539	1.04284	0.21597	0	6.66E−08	0
Farm D2	109.6386	0.000117	4.710256	0.105494	0.097014	0.00797	0
All farms together	653.77	0.000607	1.173	0.15691	0.000851	0.11596	0

Parameters obtained for the best fit when optimizing on each farm separately (first five rows) and when optimizing using only one set of parameters for all farms (last row). *α* is the coefficient of the indirect transmission, *β* is the coefficient of the cow-to-cow infection, and *δ* is the constant contribution to force of infection. Parameters *µ* and *σ* are the average and standard deviation of the latent period, and *γ* is the power of the bacterial load in the force of infection.

### All farms together analysis

In order to obtain a more robust estimate of the possible transmission models, we computed the optimal parameters, assuming all farms have equal parameters. The best-fitting ML model included indirect transmission and a constant infectivity (Figure 4B), but not the direct transmission term. The resulting model was significantly better than the null model with constant infectivity (*p* = 3.5 × 10^−7^). This can be also seen from the important contribution of the indirect transmission term to the infectivity in all farms (Figure 4B). We performed an additional optimization of the same model using a minimal least square regression with similar results (Additional files 10 and 11). We have also tested multiple alternative models, but none of them was as significant as the model with both indirect transmission and a constant infectivity term. While the direct transmission term could be removed with no effect on the resulting likelihood, removing the constant term significantly decreased the model likelihood.

The model in which transition to high shedding has a constant probability over time (fit with exponential rather than Gaussian, see Additional file 4) produced a lower likelihood and a large contribution of the constant infection pressure (*δ*) in all the models (Additional file 7). The model with the power over the total shed bacteria (and not over the bacteria shed by each cow, see Additional file 4) also produced a lower score and a large contribution of the constant term.

### Power of indirect transmission term

**Figure 5:**
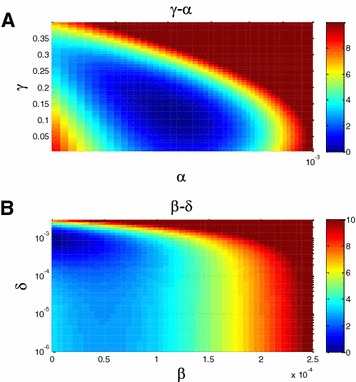
**Sensitivity analysis.** **A** Sensitivity analysis of the log likelihood as a function of infection parameters. The Z scale is minus the log of the likelihood, subtracted by the minimal cost which is the maximal likelihood. The x and y axes are the coefficient of the bacterial load in the infectivity term (*α*) and the power of the bacterial load in the infectivity term (*γ*). The optimal likelihood is indeed a minimum. **B** Sensitivity analysis as a function of infection parameters. The Z scale is minus the log of the likelihood, subtracted by the minimal cost which is the maximal likelihood. The x and y axes are the coefficient of the direct transmission term (*β*) and the constant infectivity term (*δ*). The optimal likelihood is indeed a minimum.

The highest likelihood value for the power term of the bacterial load in the indirect transmission term (*γ*) is *γ* = 0.116 (Table 1). A sensitivity analysis on the effect of changing *γ* (Figure 5A) shows that a good fit (up to a 10-fold decrease in probability) was produced in the range of values for *γ* from 0.1 to 0.35. Note that changing *γ* required a parallel change in *α* to maintain the appropriate average force of infection. This combination of *α* and *γ* resulted in a diagonal optimal cost region in the *γ*–*α* sensitivity plot. Similarly, a sensitivity analysis in the *β–δ* plane (Figure 5B, other sensitivity analysis planes appear in Additional files 12 and 13) shows again a balance between these two terms, with an optimal cost at a positive contribution of *δ* and zero contribution of *β* (Figure 5B). Thus again, in the combined model, the effect of the bacterial load is limited, and a cow shedding 10 times more than another cow has a force of infection higher by a factor of 1.2–2.2.

### Individual strain analysis

To further test the argument that the total amount of shed bacteria has a limited effect on the force of infection, we studied a farm where the spread of two strains could be studied, and tested for the optimal model for each strain. The results for each strain were qualitatively similar to the ones from the entire population regarding both the parameter values and the main contribution to force of infection (Table 2; Figure 3B). Again, two possible solutions were identified, either a direct transmission model (strain 1), or an indirect transmission with a low value of *γ* (strain 2). The value of *γ* was again between 0 and 0.47 for different models. Alternative models and cost function were tested with lower likelihoods and a larger contribution of the constant infectivity term (Additional files 14 and 15). In all these models either the contribution of the indirect term was negligible or the power *γ* was low. Thus, while differences can exist between strains, in all studied strains, the value of *γ* should be significantly lower than 1.

**Table 2:** **Best parameters obtained for multi-strain farm for the different models** **(using two types of strains).**

	Strain 1	Strain 2
Cost	113.289	89.383
*α*	4.27E−05	0.0003
*µ*	1.19	1.000007
σ	0.106	0.176
δ	0	0
γ	0.473	6.77E−08
β	0.001	0
Contribution α	0.0007	0.001
Contribution β	0.003	0
Contribution δ	0	0

### Effect of super-shedding on super-spreading and culling strategies

We have shown above that the power of shed bacteria in the infection model was low. For example, increasing the amount of bacteria shed by a factor of 10, increases the force of infection by a factor of less than 1.2–2.2. Thus, super-shedding does not lead to super-spreading. In order to test the implication of this power on culling strategies, we simulated a herd with the maximum likelihood parameters, and tested the effect of culling cows shedding above a given level. Specifically, we tested a culling strategy of removing all cows shedding above a given level, and tested the effect of this strategy on the steady state level of infected cows. We assume all cows have a limited lifespan of 3–4 years, and only removed cows from the herd when they were observed to shed above a certain level. The shedding level distributions as well as all other parameters were based on the observed farm parameters.

**Figure 6:**
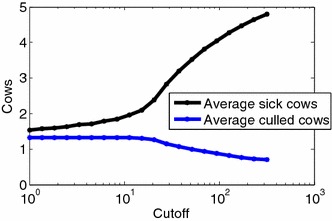
**Simulation results.** Cows which shed above the cutoff value (x axis) were culled. The average number of total culled cows (*blue*) and the average number of MAP infected cows per day (*black*) for the CFU cutoff. This number converges at cutoff = 0 to approximately *δN*. In order to significantly reduce the frequency of sick cows, a low culling level must be used. Thus, removing only super-shedders does not significantly affect the spread of the disease.

One can clearly observe that removing all high-shedders does not lead to a significant reduction in the number of infected cows in the herd (Figure 6). Moreover, even removing mildly shedding cows still does not lead to a drastic reduction in the number of infected cows. The reduction in the number of affected cows is much slower than the reduction in the total environmental bacterial load. While culling high shedders is a better strategy than removing randomly shedding cows, it is still not enough as a strategy to prevent the epidemics.

## Discussion

We have here developed a theoretical basis for the limited effect of the super-shedder paradox in MAP. The potential drastic effect of super-shedders in MAP and the resulting paradox has been studied in previous models [12]. This paradox stems from the presence of highly infectious individuals that are transiently present but do not overwhelm the system with new infections. We specifically address the issue of highly infectious individuals through the mechanism of increased probability of successful transmission. Although this question has been approached in *E.coli* O157:H7 infection in feedlot environments, the majority of studies on infectious individuals have focused on those individuals with unusually high rates of contacts [19, 45–47]. One possible conclusion from the presence of super-shedders was that they are indeed super-spreaders and that the disease dynamics are dictated by their presence. Such a conclusion has led to models of network vaccination, where the removal of hubs in the network would prevent the spread of epidemics [48, 49]. Another possible solution for the super-shedder paradox is that indirect transmission is not the main source of new infections and that direct contact between infectious and susceptible hosts is required for transmission. In such direct transmission models, if the direct transmission probability is not proportional to the level of shed bacteria, super-shedders would have no increased impact on population transmission rates. A recent study in *E.coli* O157:H7 [50] found that high shedding individuals only modestly increased the risk of transmission. They also found no evidence that environmental contamination by faeces of shedding cattle contributed to transmission over timescales longer than 3 days [50]. We here propose a similar approach where the rate of transmission is not linear with the absolute number of pathogens present in the environment, and thus super-shedders are not super-spreaders. We showed that this is indeed the case through a detailed analysis of long-term observational data on a natural spread of MAP in multiple herds, since MAP is an infectious disease with slow progression [39]. For this pathogen, it was shown here that indirect transmission determines the force of infection, but a drastic increase in the number of bacteria present due to super-shedders resulted in only a minor increase in the force of infection. Neither direct transmission nor a force of infection which is linear with the individual animal bacterial load was a better predictor of infection dynamics than models in which indirect transmission that used total bacterial burden as an indirect transmission term. Note that models which contain a direct transmission component may also explain the observed dynamics, although they are less plausible. However, the conclusion of such models would be similar to the conclusions above. We suggest here that this principle may be true for other infectious diseases where the range of free pathogen numbers produced by each infected host varies over multiple orders of magnitude [1, 6, 19, 20]. However, precise longitudinal data, similar to the data used in our analyses, will be necessary to distinguish between transmission models in other diseases.

The non-linearity of the force of infection with total bacterial load indicates that, while the number of shed bacteria may be important, its contribution to the force of infection is much less than linear. A cow which has a 1000-fold increase in MAP bacteria has at most a 10-fold increase in contribution to force of infection, and probably only a 2-fold increase (based on the most probable model). When properly generalized to include the effect of vaccination on the infection probability via each pathway, these results open the way for detailed optimization models for control strategies. For example, when environmental saturation occurs at a relatively low concentration of bacteria, removing only high shedding animals from the population may not be successful in eliminating infection. Under these conditions, the additional importance of super-shedders relative to “average” shedders is relatively minor. As mentioned before, this results appears to fit observational data in many populations where selective elimination of super-shedders does not result in elimination of infection [1, 2, 10, 14, 27]. Our findings could have major implication for control programs that previously often focused solely on identifying and eliminating high and super-shedding hosts. Note that since the force of infection is not highly affected by the amount of shed bacteria, it is hard to distinguish between direct transmission and indirect transmission which is highly sub-linear. The exact transmission routes are not well defined, but if there is indirect transmission, it is highly sub-linear.

Beyond the common effect of direct transmission, different farms had very different average forces of infection in the optimal results (Figure 4) as a result of different infection prevalence. This different force of infection can be due to the hygiene conditions in the farm or to the farm size, but also due to varying measures of infected and infectious cows, as can be observed for example in farm D over different time periods (D1 and D2).The reduction in the force of infection (the probability that a cow becomes infected in a given day from any source) can in such a case represent a decrease in the definition of newly infected cows.

MAP is an infectious disease with a slow progression. The fraction of sick cows in our data, as well as in our simulation, is 1–5% of the total number of cows. This is in good agreement with the literature [39]. Intervention programs that were used were insufficient to address long-term persistence of MAP [12].

## Additional files

10.1186/s13567-016-0323-3 **Descriptive statistics for the farms used.** Farm D was separated between samples taken before 31 Oct 1991 (D1) and samples taken after that date (D2).
10.1186/s13567-016-0323-3 **Data descriptive.** **A**. Distribution of shedding values in the real data (all the farms together). **B**. Distribution of the intervals between successive samples (all the farms together). **C**. Average number of samples per cow in each new time point.
10.1186/s13567-016-0323-3 **Notation summary.** Summary of all symbols that appear in the paper.
10.1186/s13567-016-0323-3 **Supp. Mat.** Additional information (Methods and Results).
10.1186/s13567-016-0323-3 **Schematic figure.** Description of different infectivity models. **A**. Schematic timeframe of positive and negative fecal data for one cow. “–” and “+” indicate negative and positive samples, respectively. In this example, there are two negative and two positive samples. The cow is susceptible (X) until the first negative sampling point, latent (H) between the first negative sampling and the first positive sampling, late shedding (Y1) between the first positive sampling and the second negative sampling and non-shedding infected (Y2) between the last negative sampling and its death. **B**. Example of fecal shedding levels. **C**–**E**. Different models tested for infectivity. **C**. Definition of “Infectious” cows, according to the “only Y1” model. In this model, a cow is infectious starting from the first positive sample until the last positive sample. **D**. Definition of “Infectious” cows in the “Y1+Y2” model. A cow is infectious starting from the first positive sample until 90 days after the last sample (then it is considered as dead). **E**. Definition for the “H+Y1+Y2” model. A cow with at least one positive sample is regarded as “Infectious” from birth to death (death is defined as 90 days after its last sample). **F**. Definition of “Infected”. A cow is regarded as “Infected” from the first positive sample until death (=90 days after the last sample).
10.1186/s13567-016-0323-3 **Schematic plot of Gaussian.** An example of a single Gaussian convoluted with a function which is zero before the cow was born. Such a Gaussian was used for the probability that cow *k* was infected *t* days before it started shedding.
10.1186/s13567-016-0323-3 **Contribution results for the exponential time window model.** Contribution of each term in the model to the average infectivity for each farm when using a time window representing a constant probability of starting to shed for infected cows.
10.1186/s13567-016-0323-3 **Parameters obtained for the best fit when optimizing on each farm separately.** Parameters obtained for the best fit for the different models (ML “only Y1”, ML “Y1+Y2”, ML “H+Y1+Y2” and the LSE model. In the main text only the ML “Y1+Y2” appears for all tables) when optimizing on each farm separately. *α* is the coefficient of the indirect transmission, *β* is the coefficient of the cow-to-cow infection, and *δ* is the constant contribution to force of infection. Parameters *μ* and *σ* are the average and standard deviation of the latent period, and *γ* is the power of the bacterial load in the force of infection.
10.1186/s13567-016-0323-3 **Contribution results of each infectivity term with model containing different parameters for each farm separately.** (In the main text data appears for ML “Y1+Y2” in all figures.) Contribution of each term in the models to the average infectivity in each farm, when optimization was done separately on each farm. The first term (*α*) is infection by free (externally sourced) bacteria. The second term (*β*) is cow-to-cow infection and the last term (*δ*) is a constant source. In the “only Y1” a cow is regarded as “infectious” from its first positive sample until the last positive sample. In the "Y1+Y2" model, a cow is regarded “infectious” from its first sample until its death. In the “H+Y1+Y2” model, a cow is regarded “infectious” from its birth to its death (and also if there is a positive ELISA/tissue sample).
10.1186/s13567-016-0323-3 **Parameters obtained for the best fit when optimizing on all farms together.** Parameters obtained for the best fit for all the different models when optimizing using only one set of parameters for all farms.
10.1186/s13567-016-0323-3 **Contribution results for all farms together.** Contribution of each term in the model to the average infectivity for each farm when using one set of parameters for all the farms. We have tested multiple models as in Additional file 9. In all tested model the main contribution was from the indirect transmission term, and the secondary infectivity is the constant term. There was no contribution from the direct transmission term. The contributions of each term differ among farms, since each farm has a different fraction of infected cows. However, most models yield similar results in the same farm. The different between models stems from the difference in the fraction of infectious cows.
10.1186/s13567-016-0323-3 **Sensitivity analysis.** Sensitivity analysis of the log likelihood as a function of infection parameters. The Z scale is minus the log of the likelihood, subtracted by the minimal cost which is the maximal likelihood. The x and y axes are the coefficient of the bacterial load in the infectivity term (*α*) and the coefficient of the direct transmission term (*β*). The optimal likelihood is indeed a minimum.
10.1186/s13567-016-0323-3 **Sensitivity analysis.** Sensitivity analysis of the log likelihood as a function of infection parameters. The Z scale is minus the log of the likelihood, subtracted by the minimal cost which is the maximal likelihood. The x and y axes are the coefficient of the bacterial load in the infectivity term (*α*) and the constant infectivity term (*β*). The optimal likelihood is indeed a minimum.
10.1186/s13567-016-0323-3 **Contribution results for individual strain analysis.** Contribution of each term in the model (single cow non-linear) to the average infectivity for the two strains analyzed for farm A.
10.1186/s13567-016-0323-3 **Individual strain analysis parameters.** Best parameters obtained for individual strain analysis for the different models (using two types of strains).
