# Supplementary material for: Impact of the shedding level on transmission of persistent infections in Mycobacteriumavium subspecies paratuberculosis (MAP)
Source: Vet Res. 2016 Feb 29;47:38. doi: 10.1186/s13567-016-0323-3 (PMC4772324; doi:10.1186/s13567-016-0323-3)
Supplement: Supplementary file 1 — 10.1186/s13567-016-0323-3 Descriptive statistics for the farms used. Farm D was separated between samples taken before 31 Oct 1991 (D1) and samples taken after that date (D2). [file 13567_2016_323_MOESM1_ESM.docx]

**Additional file 1** **Descriptive statistics for the farms used.** Farm D was separated between samples taken before 31 Oct 1991 (D1) and samples taken after that date (D2).

| **Farm** | **Sampled cows** | **Sampling duration (days)** | **Number of infections events (cow starts shedding)** | **Total number of sampling (shedding levels)** |
| --- | --- | --- | --- | --- |
| A | 1044 | 2429 | 45 | 6404 (72 positive) |
| B | 338 | 2276 | 12 | 2183 (15 positive) |
| C | 385 | 2613 | 37 | 2200 (54 positive) |
| D1 | 242 | 2940 | 70 | 1222 (77 positive) |
| D2 | 356 | 4084 | 34 | 1828 (42 positive) |
