# Supplementary figures and images for: Impact of the shedding level on transmission of persistent infections in Mycobacteriumavium subspecies paratuberculosis (MAP)
Source: Vet Res. 2016 Feb 29;47:38. doi: 10.1186/s13567-016-0323-3 (PMC4772324; doi:10.1186/s13567-016-0323-3)

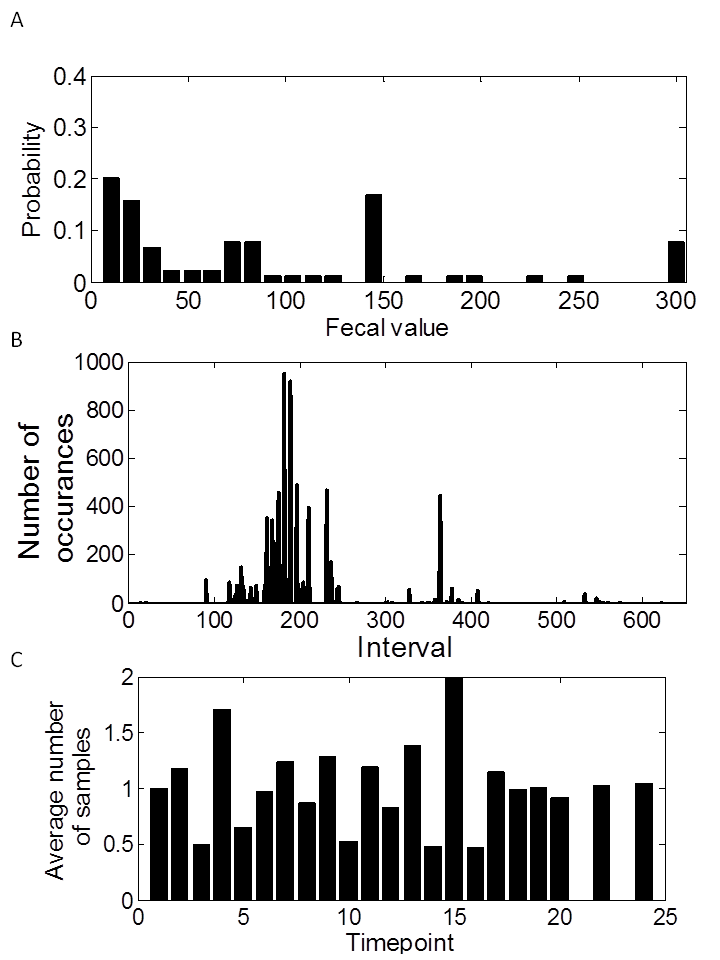

Supplement: Supplementary file 2 — 10.1186/s13567-016-0323-3 Data descriptive. A. Distribution of shedding values in the real data (all the farms together). B. Distribution of the intervals between successive samples (all the farms together). C. Average number of samples per cow in each new time point. [file 13567_2016_323_MOESM2_ESM.tif]

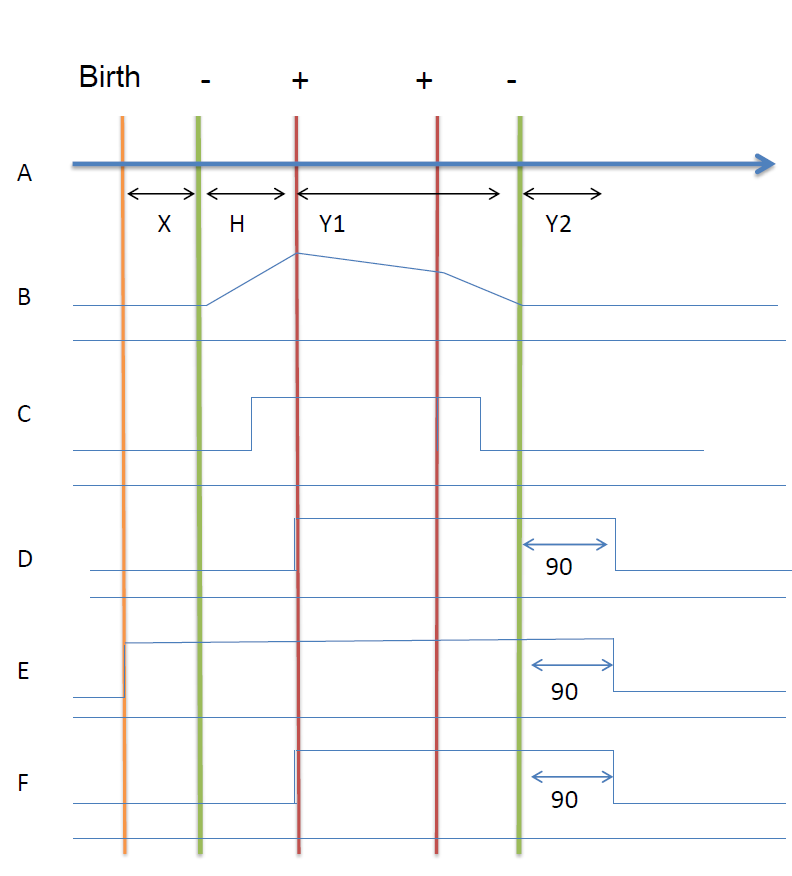

Supplement: Supplementary file 5 — 10.1186/s13567-016-0323-3 Schematic figure. Description of different infectivity models. A. Schematic timeframe of positive and negative fecal data for one cow. “–” and “+” indicate negative and positive samples, respectively. In this example, there are two negative and two positive samples. The cow is susceptible (X) until the first negative sampling point, latent (H) between the first negative sampling and the first positive sampling, late shedding (Y1) between the first positive sampling and the second negative sampling and non-shedding infected (Y2) between the last negative sampling and its death. B. Example of fecal shedding levels. C–E. Different models tested for infectivity. C. Definition of “Infectious” cows, according to the “only Y1” model. In this model, a cow is infectious starting from the first positive sample until the last positive sample. D. Definition of “Infectious” cows in the “Y1+Y2” model. A cow is infectious starting from the first positive sample until 90 days after the last sample (then it is considered as dead). E. Definition for the “H+Y1+Y2” model. A cow with at least one positive sample is regarded as “Infectious” from birth to death (death is defined as 90 days after its last sample). F. Definition of “Infected”. A cow is regarded as “Infected” from the first positive sample until death (=90 days after the last sample). [file 13567_2016_323_MOESM5_ESM.tif]

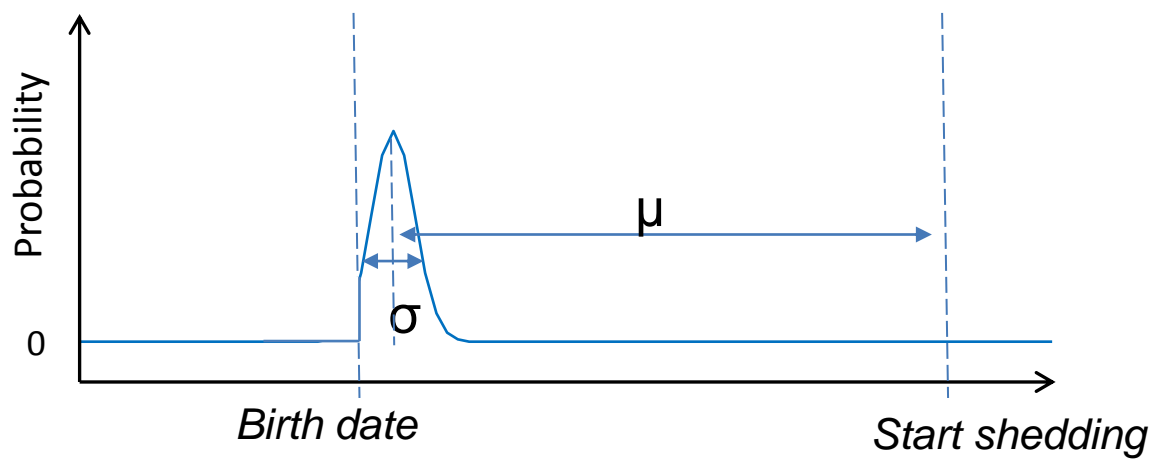

Supplement: Supplementary file 6 — 10.1186/s13567-016-0323-3 Schematic plot of Gaussian. An example of a single Gaussian convoluted with a function which is zero before the cow was born. Such a Gaussian was used for the probability that cow k was infected t days before it started shedding. [file 13567_2016_323_MOESM6_ESM.pdf]

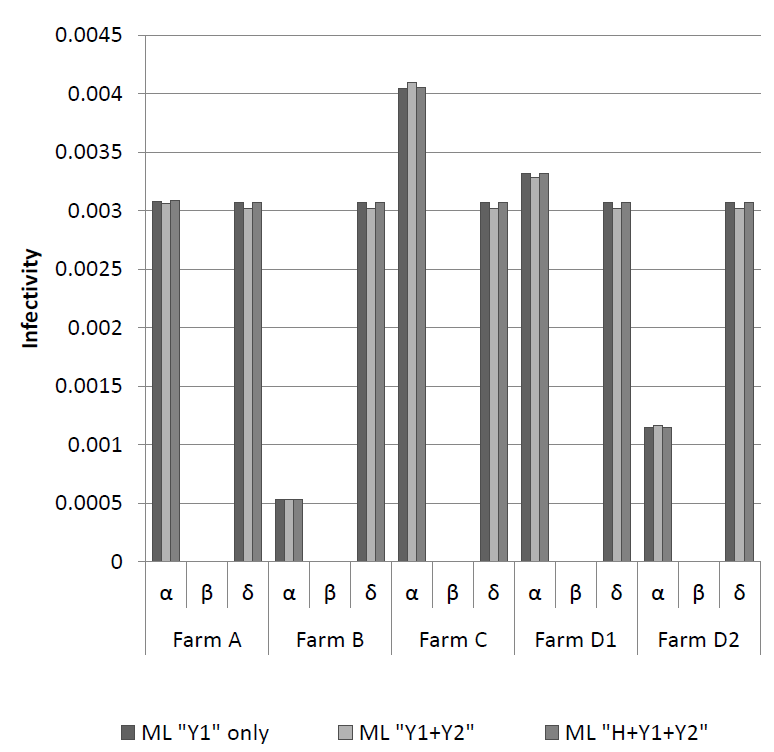

Supplement: Supplementary file 7 — 10.1186/s13567-016-0323-3 Contribution results for the exponential time window model. Contribution of each term in the model to the average infectivity for each farm when using a time window representing a constant probability of starting to shed for infected cows. [file 13567_2016_323_MOESM7_ESM.tif]

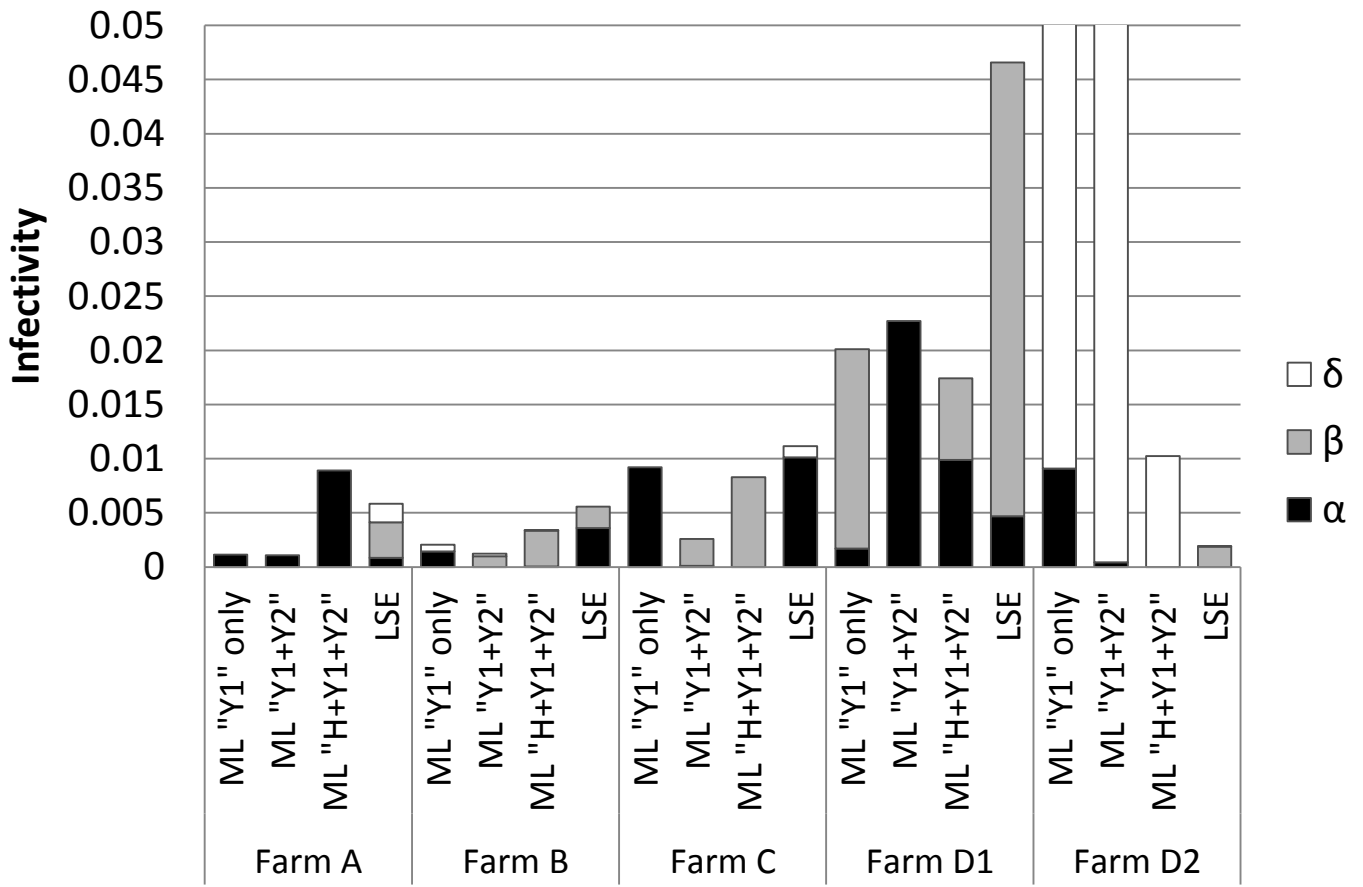

Supplement: Supplementary file 9 — 10.1186/s13567-016-0323-3 Contribution results of each infectivity term with model containing different parameters for each farm separately. (In the main text data appears for ML “Y1+Y2” in all figures.) Contribution of each term in the models to the average infectivity in each farm, when optimization was done separately on each farm. The first term (α) is infection by free (externally sourced) bacteria. The second term (β) is cow-to-cow infection and the last term (δ) is a constant source. In the “only Y1” a cow is regarded as “infectious” from its first positive sample until the last positive sample. In the "Y1+Y2" model, a cow is regarded “infectious” from its first sample until its death. In the “H+Y1+Y2” model, a cow is regarded “infectious” from its birth to its death (and also if there is a positive ELISA/tissue sample). [file 13567_2016_323_MOESM9_ESM.pdf]

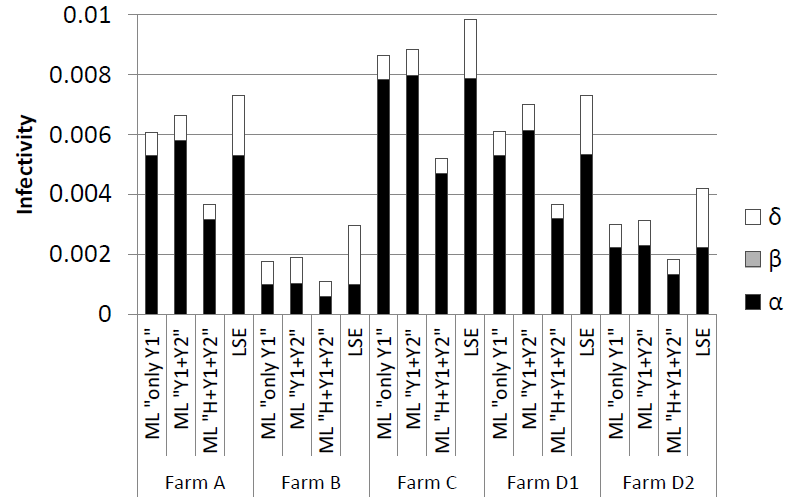

Supplement: Supplementary file 11 — 10.1186/s13567-016-0323-3 Contribution results for all farms together. Contribution of each term in the model to the average infectivity for each farm when using one set of parameters for all the farms. We have tested multiple models as in Additional file 9. In all tested model the main contribution was from the indirect transmission term, and the secondary infectivity is the constant term. There was no contribution from the direct transmission term. The contributions of each term differ among farms, since each farm has a different fraction of infected cows. However, most models yield similar results in the same farm. The different between models stems from the difference in the fraction of infectious cows. [file 13567_2016_323_MOESM11_ESM.tif]

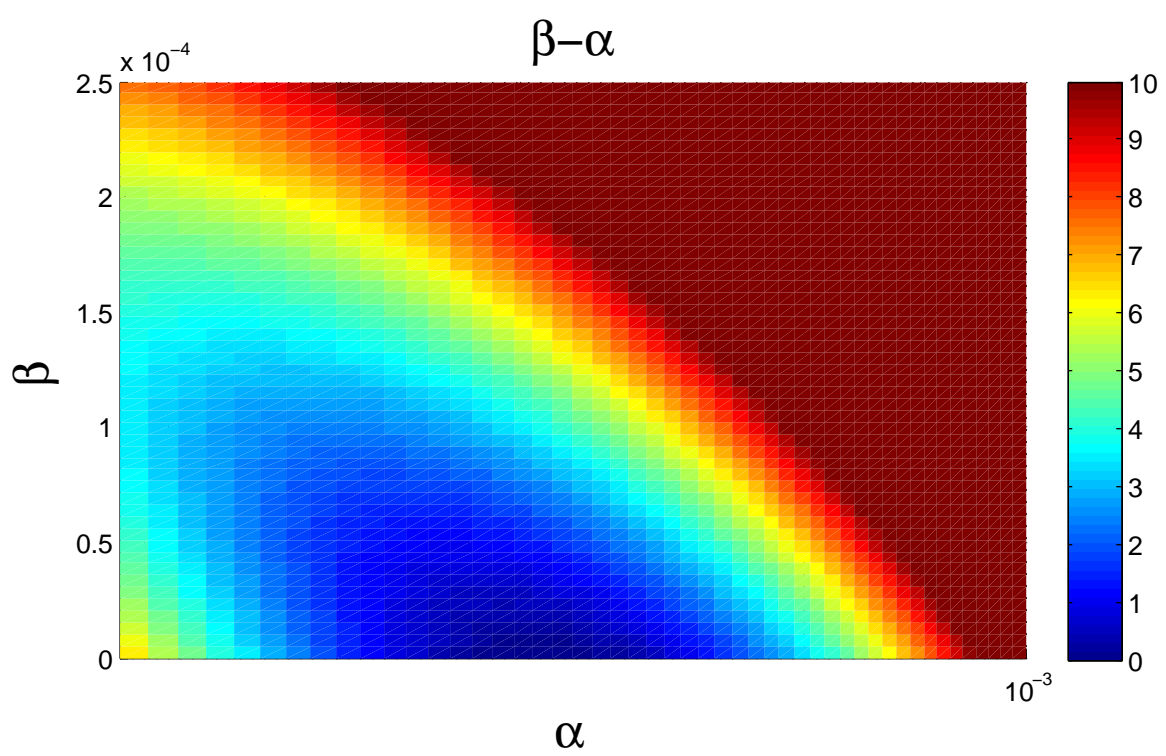

Supplement: Supplementary file 12 — 10.1186/s13567-016-0323-3 Sensitivity analysis. Sensitivity analysis of the log likelihood as a function of infection parameters. The Z scale is minus the log of the likelihood, subtracted by the minimal cost which is the maximal likelihood. The x and y axes are the coefficient of the bacterial load in the infectivity term (α) and the coefficient of the direct transmission term (β). The optimal likelihood is indeed a minimum. [file 13567_2016_323_MOESM12_ESM.pdf]

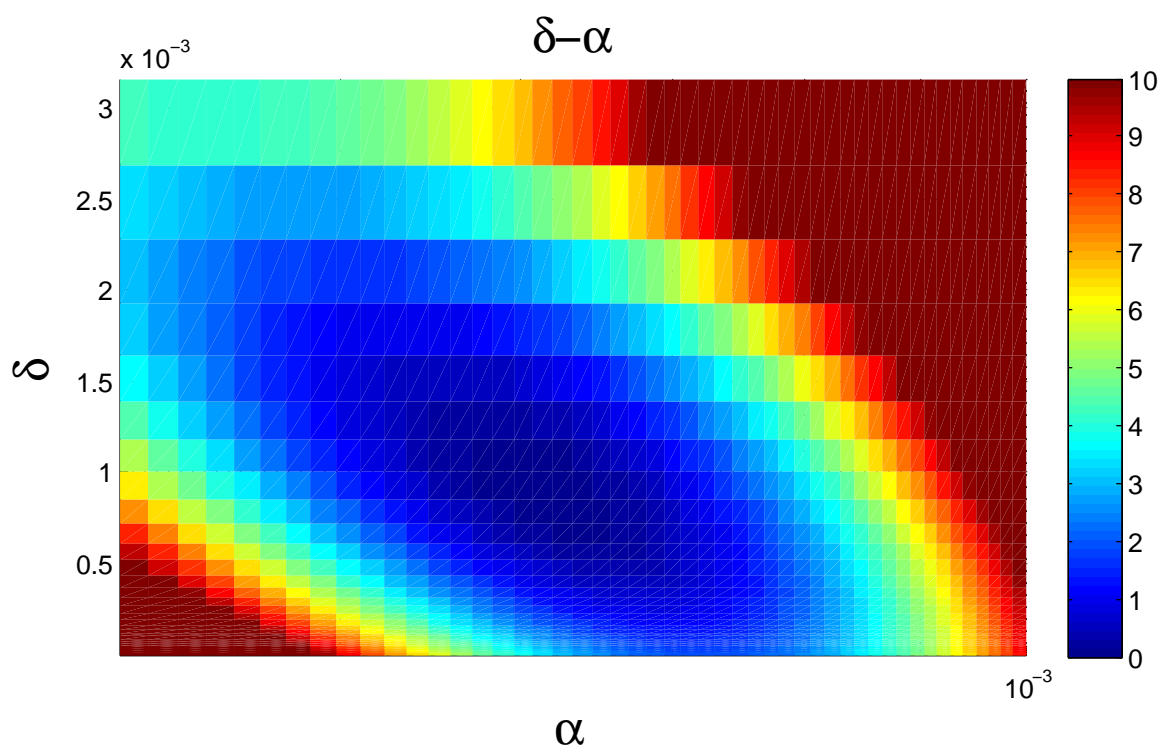

Supplement: Supplementary file 13 — 10.1186/s13567-016-0323-3 Sensitivity analysis. Sensitivity analysis of the log likelihood as a function of infection parameters. The Z scale is minus the log of the likelihood, subtracted by the minimal cost which is the maximal likelihood. The x and y axes are the coefficient of the bacterial load in the infectivity term (α) and the constant infectivity term (β). The optimal likelihood is indeed a minimum. [file 13567_2016_323_MOESM13_ESM.pdf]

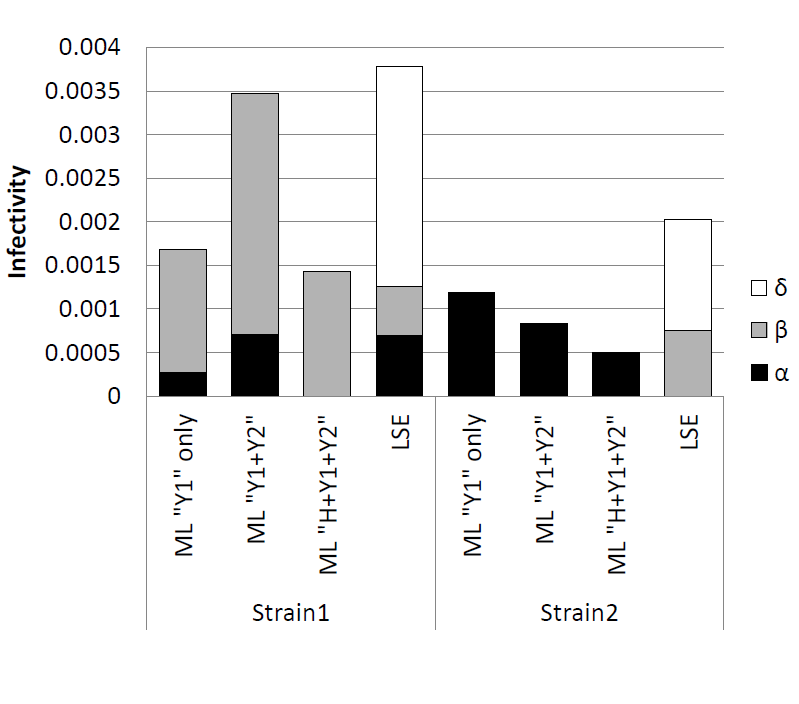

Supplement: Supplementary file 14 — 10.1186/s13567-016-0323-3 Contribution results for individual strain analysis. Contribution of each term in the model (single cow non-linear) to the average infectivity for the two strains analyzed for farm A. [file 13567_2016_323_MOESM14_ESM.tif]
