# Supplementary material for: Impact of the shedding level on transmission of persistent infections in Mycobacteriumavium subspecies paratuberculosis (MAP)
Source: Vet Res. 2016 Feb 29;47:38. doi: 10.1186/s13567-016-0323-3 (PMC4772324; doi:10.1186/s13567-016-0323-3)
Supplement: Supplementary file 3 — 10.1186/s13567-016-0323-3 Notation summary. Summary of all symbols that appear in the paper. [file 13567_2016_323_MOESM3_ESM.docx]

**Additional file 3 Notation summary**. Summary of all symbols that appear in the paper.

| Symbol | Definition |
| --- | --- |
| X | Susceptible state of a cow |
| H | Latent state of a cow |
| Y1 | Shedding state of a cow |
| Y2 | Non-shedding infected state of a cow |
| $i$ | The day that a cow was infected |
| $j*$ | The day that a cow *j* starts shedding |
| $p_{i}$ | Force of infection – probability to get infected on day i |
| $\mu$ | Average number of days between the time that a cow was infected and the time it started shedding |
| $\sigma$ | Standard deviation for the time between infection and shedding |
| $h_{ij}$ | The probability that cow *j* got infected on day *i* |
| $\delta$ | Constant infectivity term |
| $\beta$ | Cow-to-cow direct infection term |
| $\gamma$ | The power in which the amount of bacteria is raised to when dealing with the indirect infection term |
| $\alpha$ | Indirect infection term |
| $w$ | Total amount of MAP bacteria |
| $N_{i}$ | The number of cows which can get infected on day I (depends on the model – “only Y1”/”Y1+Y2”/”H+Y1+Y2”) |
| S_i_ | Number of susceptible cows on day i |
| $y_{i}$ | Number of initial shedding events on day i |
