# Supplementary material for: Impact of the shedding level on transmission of persistent infections in Mycobacteriumavium subspecies paratuberculosis (MAP)
Source: Vet Res. 2016 Feb 29;47:38. doi: 10.1186/s13567-016-0323-3 (PMC4772324; doi:10.1186/s13567-016-0323-3)
Supplement: Supplementary file 4 — 10.1186/s13567-016-0323-3 Supp. Mat. Additional information (Methods and Results). [file 13567_2016_323_MOESM4_ESM.docx]

# Additional file 4 Additional information

# Materials and methods

## Least square error (LSE) fit

We tested the fit of the data allowing different terms for the infection source, and also a least squared error (LSE) model based on minimizing the squared differences between observed and expected patterns.

For this analysis, we estimated the expected infection date of each cow based on the time it first was detected shedding MAP (Y1), and compared the number of infected cows at each date with the expected number based on calculated force of infection.

For a cow *k* that began to shed MAP on a certain day *l*, we use a Gaussian with average *µ* and a standard deviation $\sigma$ for the probability that it was infected *t* days before, as illustrated in Figure 2A. Equation 1 represents the probability that the cow *k* was infected on day *i*, where *µ* is the estimated incubation period. The part of the Gaussian representing the period before the cow was born is set to 0, preventing infection prior to birth. The remaining portion of the Gaussian is normalized to 1 (Additional file 6).

$h_{ik}=\frac{1}{\sqrt{2\pi}\sigma}e^{-\frac{\left(t-\mu\right)^{2}}{2\sigma^{2}}}=\frac{1}{\sqrt{2\pi}\sigma}e^{-\frac{\left(l-i-\mu\right)^{2}}{2\sigma^{2}}}$ (1)

A fit of a LSE model to the observations is performed by minimizing the square of the differences between the computed force of infection in the model and the predicted fraction of cows infected on a given day. At each stage, we calculated the sum of probability of infections on each day via the backwards Gaussians$\left(\tilde{h}_{i}=\sum_{k} h_{ik}\right)$. We fit the expected values of the force of infection $\left(p_{i}\right)$multiplied by the number of the susceptible cows on day *i* $\left(S_{i}\right)$ and the expected number of cows computed to get infected on day *i* as computed by the backward Gaussians (Equation 2). The total cost is a sum over the days of the squared difference. Differences between infection pressure and observed infections were not considered for the last $\mu+3\sigma$ days since only the infections which occurred previous to this point were likely to be detected during the study period.

$Err=\sum_{day\text{ }i< lastDay-\mu+3\sigma} \left(S_{i}p_{i}-\tilde{h}_{i}\right)^{2}$ (2)

Every set of parameters has a cost (the squared differences between the observed and expected patterns) for every model for every farm. When analyzing all the farms together, the total cost is the sum of the costs for each individual farm. A smaller cost means a better solution. A numerical optimization (Nelder-Mead [[36](#_ENREF_36)] with a 1000 random initial conditions) is performed in Matlab to find the optimal solution.

**Maximum likelihood fit with exponent rather than Gaussian**

We tested a model where transition to high shedding has a constant probability over time. In such a model the latency period has an exponential distribution. In the exponential model, we used an exponential function with exponent $\eta$ as in Equation 3 and calculated $p_{l}^{*}$the same way as in the Gaussian model

$Z_{l}=\frac{\sum_{i} p_{l-i}e^{-\eta(l-i)}}{\sum_{i} e^{-\eta(l-i)}}$ (3)

**Broader assumptions of the size of the infectious population**

# The cow-to-cow direct infection term ($\beta$) is proportional to either the number of shedding hosts$\left(\beta Y_{1}\right)$or the number of shedding and non-shedding $\left(\beta(Y_{1}+Y_{2})\right)$ or total number of infected cows in the herd$\left(\beta(H+Y_{1}+Y_{2})\right)$. Each of these possibilities was tested separately. Initially only Y1 individuals were allowed to contribute to infectiousness (Additional file 5D), then Y1 and Y2 (Additional file 5E), and finally all infected classes (H, Y1 and Y2) (Additional file 5F).

In the “only Y1” model, a cow is regarded as “infectious” from its first positive sample until the last positive sample. In the “Y1+Y2” model, a cow is regarded “infectious” from its first sample until its death. In the “H+Y1+Y2” a cow is regarded “infectious” from its birth to its death (and also if there is a positive ELISA/tissue sample).

# Results

## Effect of assumptions on infection progression

We tested different models of infection progression (See Additional file 5 for definitions). When comparing the best definition of infectious cows, no significant difference was observed between the optimal score of the “only Y1”, “Y1+Y2” and “H+Y1+Y2” models. There are only limited differences between the models for a few reasons: First, the value of $\gamma$ is very low, and thus the precise shedding patterns have a limited effect. Second, there is a very limited contribution of the direct infection term. Finally, cows are infectious for a long time, and the models differ only at the initial and final periods of this infectious period, which has a limited effect. In the main text sections, we used the “Y1+Y2” model.

## Alternative error definition- LSE

We tested an alternative cost function defined as the Least Square Error (LSE) of the predicted number of infection vs. the observed one (See Materials and methods above). The LSE results were similar to the ML results. The main difference is again that in some farms, a direct infection term was replaced by an indirect infection term with a power of almost 0 (Additional files 8 and 9).
