# Supplementary material for: Impact of the shedding level on transmission of persistent infections in Mycobacteriumavium subspecies paratuberculosis (MAP)
Source: Vet Res. 2016 Feb 29;47:38. doi: 10.1186/s13567-016-0323-3 (PMC4772324; doi:10.1186/s13567-016-0323-3)
Supplement: Supplementary file 8 — 10.1186/s13567-016-0323-3 Parameters obtained for the best fit when optimizing on each farm separately. Parameters obtained for the best fit for the different models (ML “only Y1”, ML “Y1+Y2”, ML “H+Y1+Y2” and the LSE model. In the main text only the ML “Y1+Y2” appears for all tables) when optimizing on each farm separately. α is the coefficient of the indirect transmission, β is the coefficient of the cow-to-cow infection, and δ is the constant contribution to force of infection. Parameters μ and σ are the average and standard deviation of the latent period, and γ is the power of the bacterial load in the force of infection. [file 13567_2016_323_MOESM8_ESM.docx]

**Additional file 8 Parameters obtained for the best fit when optimizing on each farm separately**. Parameters obtained for the best fit for the different models (ML “only Y1”, ML “Y1+Y2”, ML “H+Y1+Y2” and the LSE model. In the main text only the ML “Y1+Y2” appears for all tables) when optimizing on each farm separately. *α* is the coefficient of the indirect transmission, *β* is the coefficient of the cow-to-cow infection, and *δ* is the constant contribution to force of infection. Parameters$\mu$and $\sigma$are the average and standard deviation of the latent period, and γ is the power of the bacterial load in the force of infection.

|  |  | Cost | Alpha | Mu | Sig | Delta | Gamma | Beta |
| --- | --- | --- | --- | --- | --- | --- | --- | --- |
| Farm A | ML “Y1” only | 170.2944 | 2.05E-05 | 1.033132 | 0.1 | 0 | 0.69187 | 0 |
|  | ML “Y1+Y2” | 170.1717 | 3.49E-05 | 1 | 0.100166 | 0 | 0.551659 | 0 |
|  | ML “H+Y1+Y2” | 170.17 | 0.000306 | 1.2731 | 0.14813 | 0 | 0.53089 | 0 |
|  | LSE | 21.43604 | 2.82E-08 | 1 | 4 | 0.00171 | 1.999985 | 0.000337 |
| Farm B | ML “Y1” only | 28.17445 | 0.000616 | 4.338152 | 1.223615 | 0.000641 | 0.305108 | 0 |
|  | ML “Y1+Y2” | 28.042 | 0 | 2.8563 | 0.16409 | 0.000227 | 0.19292 | 0.00029 |
|  | ML “H+Y1+Y2” | 28.041 | 2.59E-05 | 3.2505 | 0.13827 | 6.33E-05 | 0.13289 | 0.000244 |
|  | LSE | 0.253262 | 2.48E-07 | 5.190103 | 1.779913 | 0 | 2 | 0.001189 |
| Farm C | ML “Y1” only | 114.5018 | 0.000697 | 3.113036 | 2.181362 | 0 | 9.9E-07 | 0 |
|  | ML “Y1+Y2" | 113.82 | 6.59E-06 | 1.0064 | 0.13209 | 0 | 0 | 0.000138 |
|  | ML “H+Y1+Y2” | 114.43 | 0 | 3.1928 | 1.6652 | 0 | 0.61636 | 0.000207 |
|  | LSE | 11.51278 | 0.000764 | 1 | 4 | 0.001035 | 1.13E-09 | 0 |
| Farm D1 | ML “Y1” only | 178.2993 | 0.00019 | 1.104504 | 0.155876 | 0 | 6.39E-08 | 0.002061 |
|  | ML “Y1+Y2” | 178.2996 | 0.002539 | 1.04284 | 0.21597 | 0 | 6.66E-08 | 0 |
|  | ML “H+Y1+Y2” | 177.62 | 0.001107 | 1.0059 | 0.16857 | 0 | 6.78E-21 | 0.000353 |
|  | LSE | 67.718 | 5.27E-07 | 1 | 4 | 0 | 1.8252 | 0.004683 |
| Farm D2 | ML “Y1” only | 109.6239 | 0.001527 | 5.435817 | 0.164491 | 0.1 | 0.445816 | 0 |
|  | ML “Y1+Y2” | 109.6386 | 0.000117 | 4.710256 | 0.105494 | 0.097014 | 0.00797 | 0 |
|  | ML “H+Y1+Y2” | 109.7569 | 0 | 6 | 4 | 0.010233 | 0.016148 | 0 |
|  | LSE | 1.128807 | 9.95E-10 | 4.503282 | 0.110634 | 4.55E-05 | 0.159524 | 0.000473 |
