# Supplementary material for: Impact of the shedding level on transmission of persistent infections in Mycobacteriumavium subspecies paratuberculosis (MAP)
Source: Vet Res. 2016 Feb 29;47:38. doi: 10.1186/s13567-016-0323-3 (PMC4772324; doi:10.1186/s13567-016-0323-3)
Supplement: Supplementary file 10 — 10.1186/s13567-016-0323-3 Parameters obtained for the best fit when optimizing on all farms together. Parameters obtained for the best fit for all the different models when optimizing using only one set of parameters for all farms. [file 13567_2016_323_MOESM10_ESM.docx]

**Additional file 10 Parameters** **obtained for the best fit when optimizing on all farms together.** Parameters obtained for the best fit for all the different models when optimizing using only one set of parameters for all farms.

|  | ML “only Y1” | ML “Y1+Y2” | ML “H+Y1+Y2” | LSE |
| --- | --- | --- | --- | --- |
| Cost | 654.94 | 653.77 | 654.92 | 353.28 |
| Alpha | 0.000593 | 0.000607 | 0.000356 | 0.000595 |
| Mu | 1 | 1.173 | 1.0837 | 1 |
| Sigma | 0.26833 | 0.15691 | 0.13238 | 4 |
| Delta | 0.000787 | 0.000851 | 0.000482 | 0.001978 |
| Gamma | 3.86E-08 | 0.11596 | 5.61E-08 | 1.39E-09 |
| Beta | 0 | 0 | 0 | 0 |
