# Supplementary material for: Impact of the shedding level on transmission of persistent infections in Mycobacteriumavium subspecies paratuberculosis (MAP)
Source: Vet Res. 2016 Feb 29;47:38. doi: 10.1186/s13567-016-0323-3 (PMC4772324; doi:10.1186/s13567-016-0323-3)
Supplement: Supplementary file 15 — 10.1186/s13567-016-0323-3 Individual strain analysis parameters. Best parameters obtained for individual strain analysis for the different models (using two types of strains). [file 13567_2016_323_MOESM15_ESM.docx]

**Additional file 15** **Best parameters obtained for individual strain analysis for the different models** (using two types of strains).

|  | Strain 1 |  |  |  | Strain 2 |  |  |  |
| --- | --- | --- | --- | --- | --- | --- | --- | --- |
|  | ML only Y1 | ML Y1+Y2 | ML H+Y1+Y2 | LSE | ML only Y1 | ML Y1+Y2 | ML H+Y1+Y2 | LSE |
| Cost | 112.47 | 113.29 | 112.04 | 11.40542 | 89.37693 | 89.38 | 89.56 | 3.19 |
| Alpha | 5.15E-05 | 4.27E-05 | 0 | 1.37E-05 | 0.000414 | 0.000291 | 0.000139 | 0 |
| Mu | 1.14 | 1.19 | 3.86 | 1 | 1.062627 | 1.000007 | 1.027 | 1 |
| Sigma | 0.1 | 0.11 | 0.12 | 4 | 0.243489 | 0.18 | 0.1 | 4 |
| Delta | 0 | 0 | 0 | 0.002522 | 0 | 0 | 0 | 0.001276 |
| Gamma | 0.04 | 0.47 | 0.063 | 0.735814 | 0.028306 | 6.77E-08 | 0.21 | 1.15E-07 |
| Beta | 0.000276 | 0.000445 | 3.77E-05 | 0.000109 | 1.06E-22 | 0 | 0 | 0.000233 |
| Contribution Alpha | 0.000271 | 0.000704 | 0 | 0.000694 | 0.001191 | 0.000832 | 0.000496 | 0 |
| Contribution Beta | 0.001418 | 0.002763 | 0.001433 | 0.00056 | 3.40E-22 | 0 | 0 | 0.000749 |
| Contribution Delta | 0 | 0 | 0 | 0.002522 | 0 | 0 | 0 | 0.001276 |
